# Longitudinal strain assessed by cardiac magnetic resonance correlates to hemodynamic findings in patients with severe aortic stenosis and predicts positive remodeling after transcatheter aortic valve replacement

**DOI:** 10.1007/s00392-017-1153-7

**Published:** 2017-08-14

**Authors:** Dominik Buckert, Maciej Cieslik, Raid Tibi, Michael Radermacher, Volker Rasche, Peter Bernhardt, Vinzenz Hombach, Wolfgang Rottbauer, Jochen Wöhrle

**Affiliations:** Department of Internal Medicine II, University of Ulm, Albert-Einstein-Allee 23, 89081 Ulm, Germany

**Keywords:** Cardiac magnetic resonance imaging, Strain imaging, Cardiac mechanics, Severe aortic stenosis, Transcatheter aortic valve replacement

## Abstract

**Aims:**

To assess left-ventricular strain parameters before and after transcatheter aortic valve replacement (TAVR) by feature tracking cardiac magnetic resonance imaging (FT CMR) and to correlate the findings to hemodynamic state and left-ventricular remodeling.

**Methods and results:**

Patients with symptomatic AS underwent FT CMR before and after TAVR. Patients were carefully evaluated by a comprehensive work-up including CMR, echocardiography and left and right heart catheterization. Thirty patients formed the study population. High-flow/high-gradient (HF/HG) aortic stenosis was diagnosed in 11 patients (36.7%), 6 patients (20.0%) exhibited low-flow/low-gradient AS (LF/LG) and 13 patients (43.3%) were classified to have so-called paradoxical low-flow/low-gradient (PLF/LG) AS. The HF/HG patients had a significantly reduced longitudinal strain which recovered after TAVR (−12.67 ± 4.60 to −15.46 ± 5.61%, *p* = 0.048). In the LF/LG group, an even more pronounced reduction of longitudinal strain and also an impairment of longitudinal velocity could be observed. Both parameters improved after therapy (strain: −5.06 ± 4.25 to −8.02 ± 3.28%, *p* = 0.045; velocity: 25.33 ± 9.63 to 37.13 ± 11.64 mm/s, *p* = 0.042). Patients with PLF/LG showed preserved longitudinal strain but a reduction of longitudinal velocity similar to the LF/LG group. These patients did not show a significant improvement of strain parameters after TAVR. Longitudinal velocity exhibited the highest predictive power for the identification of a low-flow state (sensitivity 75%, specificity 80%).

**Conclusion:**

Improvement of longitudinal strain parameters after TAVR is dependent on the initial hemodynamically defined AS subgroup.

## Introduction

Aortic stenosis (AS) is one of the most common valvular pathologies in western populations, especially in the elderly [1]. Once severe AS causes symptoms, indication for aortic valve replacement is given [2]. In patients at high surgical risk, transcatheter aortic valve replacement (TAVR) has been shown to be superior to surgical valve replacement [3–6].

The current definition of severe AS comprises either an aortic valve area (AVA) ≤1.0 cm^2^, or a mean pressure gradient (MPG) ≥40 mmHg or a peak velocity ≥4 m/s [2, 7]. Since MPG and peak velocity are highly dependent on flow across the valve, premise of this definition is a hemodynamic situation in which a normal flow and usually a normal left-ventricular ejection fraction (LVEF) are given [8]. Patients exhibiting these features are called to have ‘high-flow/high-gradient AS (HF/HG)’ [7, 8]. However, there are specific subsets of patients suffering from severe AS in which the hemodynamic situations are different. In patients with reduced LVEF, either caused by the AS itself or other cardiac pathologies, a ‘low-flow/low-gradient AS (LF/LG)’ may be present due to decreased stroke volume and low flow across the valve [9]. There is a third important subset of patients in which the chronic pressure overload caused by longstanding AS leads to a progressive concentric hypertrophy and a severe diastolic dysfunction [10]. These patients also show decreased stroke volumes and flow despite LVEF is preserved and, thus, have a ‘paradoxical low-flow/low-gradient AS (PLF/LG)’ [11]. Correct diagnosis and identification of the specific AS pathology are of great importance, since outcomes and prognosis of the particular subpopulations and their response to aortic valve replacement differ [12].

Strain imaging is an emerging technique for the non-invasive evaluation of global and regional left ventricular function [13]. Its ability to characterize contractility patterns in different hemodynamic situations has been proven, especially for longitudinal strain [14]. Accordingly, it was successfully used to assess functional properties of different AS pathologies as well as the specific changes after valve replacement [15]. In most of the studies forming the evidence base, strain values were derived from 2D speckle-tracking echocardiography. Though this imaging technique is highly accepted and widely available, it exhibits several limitations such as relative low inter-reader and intra-reader reproducibility and the need for an appropriate ‘acoustic window’ [16, 17].

With its high spatial resolution, good image contrast, lack of ionizing radiation and favorable reproducibility, cardiac magnetic resonance imaging (CMR) plays an increasing role in the diagnostic management of patients suffering from valve pathologies [18, 19]. It is considered the gold standard for the evaluation and quantification of left ventricular functional parameters such as volumes and LVEF [20–22]. Recently, feature tracking (FT) techniques have been introduced to derive strain parameters from conventional CMR cine images [23, 24].

The objective of our study was to assess left ventricular longitudinal strain parameters by FT CMR before and after TAVR and to correlate the findings to the underlying AS pathology.

## Methods

### Study populations

Patients with symptomatic severe AS undergoing TAVR were enrolled from 2014 to 2015 [25]. All patients were considered eligible unless they exhibited predefined exclusion criteria such as cardiac or respiratory instability, metal implants or devices unsuitable for CMR, concomitant limiting disease, allergy against gadolinium-based contrast agents or severely impaired renal function (estimated glomerular filtration rate <30 ml/min). An age- and sex-matched healthy control population was derived from a database formed within another project.

All TAVR patients were carefully evaluated concerning other cardiac diseases such as coronary heart disease, relevant other valvular heart disease, hypertrophic or dilative cardiomyopathy, diastolic dysfunction and inflammatory heart diseases. For the healthy controls, these conditions were rigorously ruled out. The study was approved by the institutional ethics committee (clinicaltrials.gov: NCT02162069). Written informed consent was obtained from every patient as well as from the healthy controls.

### Hemodynamic evaluation

All patients received a comprehensive diagnostic work-up prior to TAVR including CMR, transthoracic echocardiography and hemodynamic left and right heart catheterization. Pressure gradients across the aortic valve were invasively assessed by simultaneous measurements in the left ventricle and aorta. Hemodynamic evaluation included the invasive and non-invasive measurement of pressure gradients, pulmonary artery pressures, systemic pressures, stroke volumes, cardiac output and cardiac power index [26]. A ‘low-flow state’ was defined as a left-ventricular stroke index (LVSVi) ≤35 ml/m^2^ and/or a cardiac index (Ci) ≤3.0 l/min [27]. These parameters can be assessed by invasive measurement as well as non-invasively by CMR and echo. Every patient was carefully evaluated taking into account the results of every modality as well as supporting features (left-ventricular volumes, diastolic dysfunction) in case of conflicting results. Plausibility of the results of each modality was rated individually for each patient. In conclusion of all findings, hemodynamic state of each patient was defined according to the current guidelines and recommendations (HF/HG, LF/LG, PLF/LG) [26–28]. Assignment to a specific AS subgroup was done before strain assessment was performed in order to avoid bias.

### CMR examination

All patients received CMR examinations within 5 days before and at 3 months after TAVR. CMR imaging was performed on a 1.5-T whole body clinical magnetic resonance scanner (Achieva 1.5T, Philips Medical Systems, Best, Netherlands) using a 32-channel phased-array receiver coil. CMR examinations were carried out in concordance with current guidelines [29, 30]. A steady-state free precession sequence (SSFP, repetition time 3.4 ms, echo time 1.7 ms, voxel size 1.6 × 1.6 mm, flip-angle *α* 55°, slice thickness 8 mm, acquisition in end-expiratory breath-hold, 32 cardiac phases) was used for functional imaging of the left and right ventricle in long- and short-axis orientation.

### CMR analysis

Two experienced readers, blinded to patient history and hemodynamic findings, performed offline image analysis. Epi- and endocardial contours were drawn manually in the long- and short-axis-oriented SSFP-images. Basic functional and strain parameters (strain, systolic strain rate, displacement, systolic velocity) were derived from the SSFP cine images using the dedicated software cvi^42®^ (Version 5.2, Circle Cardiovascular Imaging, Calgary, Canada). Strain parameters were assessed globally in longitudinal, radial and circumferential orientation for the left ventricle according to the current recommendations [31, 32]. A healthy control population was set up for the validation of the used approach and the yielded results.

### Statistical analysis

To test the correlation between two categorical classification factors, the Chi squared test was applied. Continuous variables were tested for normal distribution by the D’Agostino–Pearson test. Variables with normal distribution were reported as mean ± standard deviation and a two-tailed *t* test (either for paired or independent samples) was used for comparison. Variables without normal distribution were reported as median with percentiles and compared by the Mann–Whitney *U* rank sum test. Intra-class correlation coefficient and inter-rater agreement were assessed to evaluate reproducibility and inter-rater reliability of strain imaging analyzes. To determine the accuracy of the strain-based low-flow state classification, a receiver operating characteristic (ROC) curve analysis was performed. A *p* value <0.05 was considered significant. Statistical analyses were performed using commercially available software (Stata 13, College Station, USA, MedCalc, Mariakerke, Belgium).

## Results

### Study populations

Thirty patients suffering from severe AS were enrolled in this study. Mean age was 78.8 ± 5.9 years, 50.0% were men (*n* = 15). Mean NYHA class before TAVR was 3.1 ± 0.5. The score of the Society of Thoracic Surgeons (STS) for mortality was 4.7 ± 3.0% [33]. The EURO II score was 5.4 ± 3.8% [34]. Clinical baseline characteristics are depicted in Table 1. The control group consisted of 40 individuals (20 men and 20 women) at a mean age of 74.4 ± 2.8 years. None of the patients had paraprosthetic aortic regurgitation more than ‘trace’ or required permanent pacemaker stimulation after TAVR.

**Table 1:** Baseline characteristics

	Total cohort (*n* = 30)	
Age (years)	78.8 ± 5.9	Mean ± SD
Sex (men)	15 (50.0)	N, (%)
BMI (kg/m^2^)	27.4 ± 5.1	Mean ± SD
Hypertension	29 (96.7)	N, (%)
Hyperlipoproteinaemia	24 (80.0)	N, (%)
Diabetes mellitus	11 (36.7)	N, (%)
Coronary artery disease	24 (80.0)	N, (%)
Atrial fibrillation	13 (43.3)	N, (%)
NYHA class	3.1 ± 0.6	Mean ± SD
EURO score II	5.4 ± 3.8	Mean ± SD
STS-Score	4.7 ± 3.0	Mean ± SD

*BMI* body mass index, *NYHA* New York Heart Association, *STS* Society of Thoracic Surgeons

### Hemodynamic evaluation

An HF/HG AS was observed in 11 patients (36.7%). Six subjects (20.0%) were categorized in the LF/LG group.

A PLF/LG situation was diagnosed in 13 cases (43.3%). In the HF/HG group, MPG was 47.5 ± 16.1 mmHg, LVSVi was 45.3 ± 6.4 ml/m^2^, Ci was 3.3 ± 0.58 l/min and AVA was 0.56 ± 0.13 cm^2^. Patients in the LF/LG group showed a severely reduced LVEF with 26.3 ± 7.2%, an MPG of 28.8 ± 9.5 mmHg, an LVSVi of 30.4 ± 4.9 ml/m^2^, a Ci of 2.5 ± 0.71 l/min and an AVA of 0.80 ± 0.14 cm^2^. In the PLF/LG group, a preserved LVEF could be observed with 63.7 ± 8.4%. These patients exhibited an MPG of 26.1 ± 5.7 mmHg, an LVSVi of 32.7 ± 5.9 ml/m^2^, a Ci of 2.7 ± 0.47 l/min and an AVA of 0.77 ± 0.22 cm^2^. In the PLF/LG group, 7 patients exhibited only mild hypertrophy (≤0.8 g/ml) as defined by the left-ventricular volume/mass ratio. Five of them (71.4%) were women. Table 2 shows the hemodynamic characteristics of the total cohort and the different AS entities.

**Table 2:** Hemodynamic characteristics

	Total cohort	HF/HG	LF/LG	PLF/LG
LVEF (%)	56.7 ± 18.4	65.0 ± 13.2	26.3 ± 7.2	63.7 ± 8.4
LVEDVi (ml/m^2^)	79.5 ± 30.9	73.0 ± 21.3	123.3 ± 36.1	64.8 ± 12.43
LVSVi (ml/m^2^)	40.0 ± 8.3	45.3 ± 6.4	30.4 ± 4.9	32.72 ± 5.9
Ci	2.9 ± 0.66	3.3 ± 0.58	2.5 ± 0.71	2.7 ± 0.47
RVEF (%)	60.4 ± 12.2	67.8 ± 8.1	46.8 ± 13.0	60.3 ± 9.7
RVEDVi (ml/m^2^)	68.7 ± 15.4	62.6 ± 11.0	80.5 ± 22.6	68.3 ± 12.5
MPG (mmHg)	34.0 ± 14.6	47.5 ± 16.1	28.8 ± 9.5	26.1 ± 5.7
AVA (cm^2^)	0.69 ± 0.21	0.56 ± 0.13	0.80 ± 0.14	0.77 ± 0.22
LVEDP (mmHg)	20.7 ± 6.2	19.7 ± 6.7	26.5 ± 2.1	20.3 ± 6.1

Values are mean ± standard deviation

*AVA* aortic valve area, *CI* cardiac index, *HF/HG* high-flow/high-gradient aortic stenosis, *LF/LG* low-flow/low-gradient aortic stenosis, *LVEDVi* left-ventricular end-diastolic volume index, *LVEDP* left-ventricular end-diastolic pressure, *LVEF* left ventricular ejection fraction, *LVSVi* left-ventricular stroke volume index, *MPG* mean pressure gradient, *PLF/LG* paradoxical low-flow/low-gradient aortic stenosis, *RVEDVI* right-ventricular end-diastolic volume index, *RVEF* right-ventricular ejection fraction

### Presence of late gadolinum enhancement, coronary artery disease and concomitant valve pathologies

Late gadolinium enhancement was assessed in the initial CMR study. Fourteen patients (46.7% of the total study group) were LGE positive. Eleven of them showed LGE patterns consistent with fibrosis (36.7%). Though there was no statistically significant difference concerning distribution of LGE throughout the AS subgroups, patients predominantly tended to be LGE positive in the HF/HG and LF/LG group (HF/HG: *n* = 5, 45.5%; LF/LG: *n* = 4, 66.7%; PLF/LG: *n* = 2, 15.4%; *p* = 0.07 for distribution). A relevant coronary artery disease was present in *n* = 24 (80%) of the total study cohort with no significant difference concerning the distribution between the subgroups (HF/HG: *n* = 8, 72.7%; LF/LG: *n* = 5, 83.3%; PLF/LG: *n* = 11, 84.6%; *p* = 0.47). In Table 3, frequencies of late gadolinium enhancement and coronary artery disease are depicted. None of the patients had a concomitant aortic or mitral valve regurgitation higher than grade 1.

**Table 3:** Presence of coronary artery disease and late gadolinium enhancement

	Total cohort	HF/HG	LF/LG	PLF/LG
Presence of Coronary artery disease (*n*, %)	24 (80.0)	8 (72.7)	5 (83.3)	11 (84.6)
Late gadolinium enhancement (*n*, %)	14 (46.7)	7 (54.5)	6 (100)	2 (15.4)
Consistent with fibrosis	11 (36.7)	5 (45.5)	4 (66.7)	2 (15.4)

*HF/HG* high-flow/high-gradient aortic stenosis, *LF/LG* low-flow/low-gradient aortic stenosis, *PLF/LG* paradoxical low-flow/low-gradient aortic stenosis

### Strain imaging before TAVR

Longitudinal strain parameters were assessed with excellent reproducibility and reliability as detailed in Table 4.

**Table 4:** Intraclass correlation and inter-reader agreement of longitudinal strain

	Intra-class correlation	Inter-reader agreement
Longitudinal		
Peak strain (%)	0.94 CI [0.70; 0.98]	Kappa: 0.94; Standard error: 0.02 CI [0.90; 0.97]
Peak strain rate systolic (%/s)	0.90 CI [0.73; 0.95]	Kappa: 0.83 Standard error: 0.05 CI [0.73; 0.93]
Peak displacement (mm)	0.98 CI [0.96; 0.99]	Kappa: 0.98 Standard error: 0.01 CI [0.96; 0.99]
Peak velocity (mm/s)	0.98 CI [0.95; 0.99]	Kappa: 0.95 Standard error: 0.03 CI [0.90; 1.00]

*CI* confidence interval

**Fig. 1:**
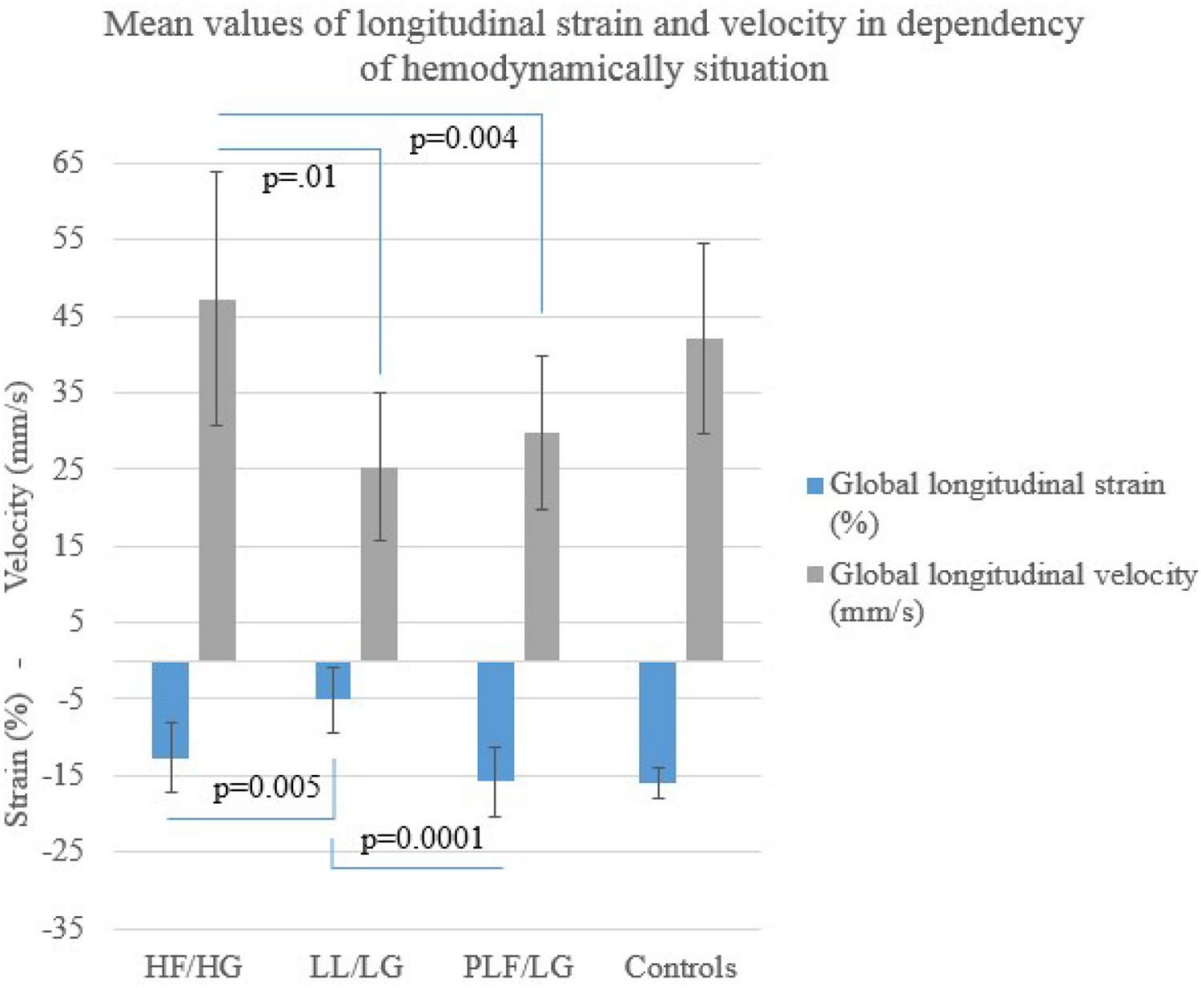
Global longitudinal strain and global longitudinal velocity in dependency of hemodynamic subgroups. In comparison to the HF/HG group, a significant reduction of strain and velocity could be observed for the LF/LG group. Patients in the PLF/LG group showed strain comparable to the HF/HG group but a reduction in velocity similar to the LF/LG group (*HF/HG* high-flow/high-gradient aortic stenosis, *LF/LG* low-flow/low-gradient aortic stenosis, *PLF/LG* paradoxical low-flow/low-gradient aortic stenosis)

Table 5 shows the results of strain analysis for the total cohort, the AS subgroups and the healthy controls. The HF/HG patients showed a slightly but statistically significant reduced global longitudinal strain in comparison to the controls (−12.67 ± 4.60 vs. −15.91 ± 1.96%, *p* = 0.001). Displacement and velocity were not significantly different. In comparison to the controls and the HF/HG group, the subjects of the LF/LG group showed not only a significant reduction of strain (comparison to HF/HG: −5.06 ± 4.25 vs. −12.67 ± 4.60%, *p* = 0.005; comparison to controls: −5.06 ± 4.25 vs. −15.91 ± 1.96%, *p* < 0.0001) but also of longitudinal velocity (comparison to HF/HG: 25.33 ± 9.63 vs. 47.24 ± 16.63 mm/s, *p* = 0.01; comparison to controls: 25.33 ± 9.63 mm/s vs. 42.02 ± 12.39, *p* = 0.003). The PLF/LG group showed preserved strain (comparison to controls: −15.80 ± 4.56 vs. −15.91 ± 1.96%, *p* = 0.90) but a reduction in velocity comparable to the LF/LG group (comparison to controls: 29.76 ± 9.98 mm/s vs. 42.02 ± 12.39, *p* = 0.002). Mean strain and velocity values for the subgroups and controls are depicted in Fig. 1.

**Table 5:** Strain imaging before transcatheter aortic valve replacement

	Total cohort	HF/HG	LG/LG	PLF/LG	Controls
Longitudinal					
Peak strain (%)	−12.61 ± 5.58	−12.67 ± 4.60	−5.06 ± 4.25	−15.80 ± 4.56	−15.91 ± 1.96
Peak strain rate systolic (%/s)	79.20 ± 37.39	97.79 ± 47.93	42.69 ± 14.53	81 ± 28.48	91.38 ± 23.54
Peak displacement (mm)	3.62 ± 1.42	3.61 ± 1.21	2.40 ± 1.91	3.98 ± 1.21	3.30 ± 1.28
Peak velocity (mm/s)	35.30 ± 16.01	47.24 ± 16.63	25.33 ± 9.63	29.76 ± 9.98	42.02 ± 12.39
Radial					
Peak strain (%)	29.20 ± 13.50	30.94 ± 10.09	12.05 ± 7.37	35.65 ± 11.76	47.16 ± 11.37
Peak strain rate systolic (%/s)	221.46 ± 95.62	248.46 ± 78.26	91.69 ± 64.79	258.51 ± 68.33	291.15 ± 93.07
Peak displacement (mm)	4.03 ± 2.03	4.00 ± 2.17	2.07 ± 1.31	4.96 ± 1.56	6.84 ± 0.87
Peak velocity (mm/s)	26.46 ± 10.79	29.95 ± 10.98	12.53 ± 6.10	29.95 ± 6.65	40.77 ± 8.61
Circumferential					
Peak strain (%)	−10.37 ± 9.02	−9.13 ± 11.78	−3.00 ± 4.47	−14.83 ± 4.75	−18.46 ± 2.41
Peak strain rate systolic (%/s)	−93.02 ± 48.78	−97.36 ± 48.24	−27.00 ± 18.24	−119.84 ± 26.38	−105.13 ± 25.40
Peak displacement (deg)	0.04 ± 1.79	−0.36 ± 2.06	0.73 ± 1.61	0.03 ± 1.70	−0.11 ± 0.15
Peak velocity (deg/s)	−4.49 ± 21.91	−4.91 ± 23.80	6.22 ± 21.84	−9.08 ± 20.23	−1.29 ± 0.95

Values are mean ± standard deviation

*HF/HG* high-flow/high-gradient aortic stenosis, *LF/LG* low-flow/low-gradient aortic stenosis, *PLF/LG* paradoxical low-flow/low-gradient aortic stenosis

Deformation parameters in radial and circumferential orientation were significantly reduced for all AS subgroups in comparison to the healthy control group (Table 5). A correlation between radial and circumferential strain and hemodynamic state could not be observed.

**Fig. 2:**
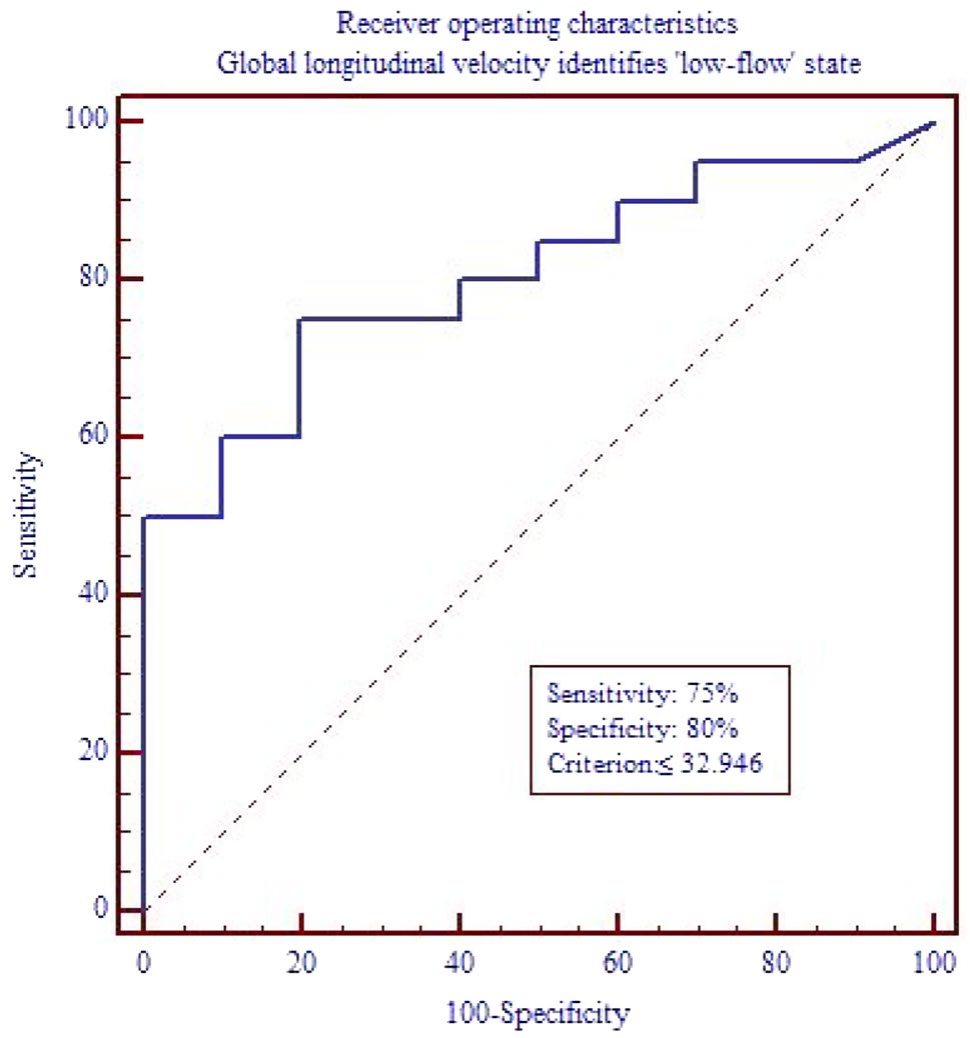
Receiver operating characteristics analysis. By the use of a cutoff criterion of ≤32.946 mm/s, a ‘low-flow’ state could be predicted by global longitudinal velocity with a sensitivity of 75% and a specificity of 80%

To assess the predictive power of global longitudinal velocity for the identification of a ‘low-flow’ state, an ROC curve analysis was performed. The result is depicted in Fig. 2. By the use of a cutoff criterion of ≤32.946 mm/s, global longitudinal velocity correctly predicted a ‘low-flow’ situation with a sensitivity of 75% and a specificity of 80%.

### Changes after TAVR

Tables 6 and 7 show changes in selected clinical variables (Table 6) and strain parameters (Table 7) in dependency of the initial AS subgroup. For the total study cohort, NT-proBNP before and after TAVR was 3804.1 ± 3909.4 and 3339.4 ± 3533.6 pg/ml (*p* = 0.36), respectively. Though statistical significance was not reached, NT-proBNP levels were lower after TAVR for the HF/HG and LF/LG groups (HF/HG: 3823.9 ± 3966.3 vs. 2669.7 ± 2163.26, *p* = 0.28; LF/LG: 6822.5 ± 4844.9 vs. 6580.3 ± 6146.5 pg/ml, *p* = 0.85). In the PLF/LG group, NT-proBNP levels were higher after the intervention (PLF/LG: 2056.6 ± 2474.7 vs. 2253.0 ± 2033.0 pg/ml, *p* = 0.65).

**Table 6:** Changes after transcatheter aortic valve replacement in dependency of hemodynamic situation

	Before TAVR	After TAVR	Change (paired differences)	
High-flow/high-gradient aortic stenosis				
NYHA class	3.4 ± 0.55	2.0 ± 0.71	−1.4 ± 0.89	0.025
Left-ventricular ejection fraction (%)	66.8 ± 12.41	69.3 ± 8.77	2.5 ± 13.47	0.572
NT-proBNP (pg/ml)	3823.9 ± 3966.3	2669.7 ± 2163.26	1154.2 ± 2769.6	0.277
Low-flow/low-gradient aortic stenosis				
NYHA class	3.31 ± 0.48	2.08 ± 0.64	−1.23 ± 0.83	0.004
Left-ventricular ejection fraction (%)	26.3 ± 7.23	35.5 ± 13.69	9.17 ± 7.22	0.027
NT-proBNP (pg/ml)	6822.5 ± 4844.9	6580.3 ± 6146.5	242.3 ± 2381.3	0.852
Paradoxical low-flow/low-gradient aortic stenosis				
NYHA class	3.29 ± 0.49	2.29 ± 4.9	−1.00 ± 0.82	0.0018
Left-ventricular ejection fraction (%)	63.69 ± 8.40	65.77 ± 9.68	2.08 ± 5.51	0.200
NT-proBNP (pg/ml)	2056.6 ± 2474.7	2253.0 ± 2033.0	−196.3 ± 1086.9	0.650

Values are reported as mean ± standard deviation or *n*, %

*TAVR* transcatheter aortic valve replacement

**Table 7:** Changes in strain after transcatheter aortic valve replacement in dependency of hemodynamic situation

	Before TAVR	After TAVR	Change (paired differences)	*p*
High-flow/high-gradient aortic stenosis				
Longitudinal				
Peak strain (%)	−12.67 ± 4.60	−15.46 ± 5.61	−3.55 ± 4.58	0.048
Peak strain rate systolic (%/s)	101.56 ± 52.72	105.49 ± 24.36	3.93 ± 72.99	0.876
Peak displacement (mm)	3.44 ± 1.17	5.09 ± 1.46	1.65 ± 1.63	0.016
Peak velocity (mm/s)	46.31 ± 18.07	47.33 ± 12.87	1.02 ± 25.48	0.907
Low-flow/low-gradient aortic stenosis				
Longitudinal				
Peak strain (%)	−5.06 ± 4.25	−8.02 ± 3.28	−2.96 ± 2.73	0.045
Peak strain rate systolic (%/s)	41.98 ± 16.68	50.43 ± 15.83	8.45 ± 21.01	0.480
Peak displacement (mm)	2.40 ± 1.91	2.72 ± 1.79	0.32 ± 1.39	0.601
Peak velocity (mm/s)	25.33 ± 9.63	37.13 ± 11.64	11.80 ± 10.65	0.042
Paradoxical low-flow/low-gradient aortic stenosis				
Longitudinal				
Peak strain (%)	−15.80 ± 4.56	−14.85 ± 5.30	0.96 ± 4.94	0.498
Peak strain rate systolic (%/s)	81.0 ± 28.48	88.65 ± 21.56	7.65 ± 21.55	0.224
Peak displacement (mm)	3.98 ± 1.21	3.16 ± 1.31	−0.82 ± 1.34	0.046
Peak velocity (mm/s)	29.76 ± 9.98	29.47 ± 9.58	−0.29 ± 9.45	0.914

Values are reported as mean ± standard deviation or *n*, %

*TAVR* transcatheter aortic valve replacement

Patients in the HF/HG group additionally showed a significant increase in global longitudinal strain (−12.67 ± 4.60 to −15.46 ± 5.61%, *p* = 0.048) and longitudinal displacement (3.44 ± 1.17 to 5.09 ± 1.46 mm, *p* = 0.016). These patients thus reached values comparable to the healthy controls (comparison of longitudinal strain after TAVR to controls: −15.46 ± 6.61 vs. −15.91 ± 1.96%, *p* = 0.67). A significant increase in longitudinal strain could also be observed for the LF/LG group (−5.06 ± 4.25 to −8.02 ± 3.28%, *p* = 0.045). These patients also exhibited a significant improvement in longitudinal velocity (25.33 ± 9.63 mm/s to 37.13 ± 11.64, *p* = 0.042) and LVEF (26.3 ± 7.23 to 35.5 ± 13.69, *p* = 0.027). There was no improvement in strain parameters for patients of the PLF/LG group.

## Discussion

We are able to show a strong correlation between CMR-derived strain patterns and the hemodynamic situation in severe AS patients.

Patients with HF/HG AS showed only a mild reduction of longitudinal strain and preserved longitudinal velocity. In the LF/LG group, reduced strain and velocity could be observed. Patients in the PLF/LG group exhibited reduced longitudinal velocity despite preserved longitudinal strain. Global longitudinal velocity reliably could identify a ‘low-flow’ state, irrespective of other hemodynamic or morphologic findings. By evaluation of strain parameters, specific responses following TAVR concerning left-ventricular remodeling could be demonstrated. For this purpose, an emerging technique and dedicated post-processing software were validated.

Comprehensive cardiac imaging is very helpful to judge the complex situation in severe AS patients. A CMR study including 91 patients could demonstrate varying patterns of left-ventricular hypertrophy and remodeling, unrelated to the severity and duration of AS [35]. The findings reflect different left-ventricular compensatory mechanisms in response to aortic valve narrowing and confirms CMR as gold standard for the assessment of left-ventricular function. CMR-derived strain may strengthen the relevance of this modality in the diagnostic work-up of AS patients. We were able to show that CMR strain imaging offers the possibility to non-invasively assess the hemodynamic situation of individual patients. Each hemodynamic subgroup was characterized by software-specific values. This may facilitate diagnosis especially for patients with a PLF/LG situation. This is of special interest, since this challenging diagnosis accounts for approximately one-third of the total severe AS population [8, 12]. Currently, a ‘low-flow’ state is defined as LVSVi ≤35 ml/m^2^ [25]. Calculation of this parameter depends on the formula used and often falls close to the 35 ml/m^2^ cutoff, which makes correct diagnosis of ‘low-flow’ still difficult [27, 28]. It could be demonstrated that echocardiographic assessment of left-ventricular longitudinal function by Doppler-derived mitral annular peak velocity may provide helpful additional information in this situation [36]. Nevertheless, low reproducibility and the need for an appropriate ‘acoustic window’ limit the widespread use of echocardiographic techniques in clinical routine [16, 17]. FT CMR-derived assessment of global longitudinal strain and velocity with a high intra- and inter-reader reproducibility may be more beneficial in those patients.

Several studies have focused on left-ventricular remodeling and its correlation to outcome after aortic valve replacement and reported conflicting results [37, 38]. A study examining 50 patients undergoing either surgical or transcatheter aortic valve replacement showed a significant decrease in right and left ventricular volumes and muscle masses, as well as a little but significant increase in LVEF [36]. In contrast, another CMR study examining a small population including 27 patients before and after TAVR did not report significant changes in LVEF [38]. It is of note that most of the patients included in the first study already exhibited a reduced LVEF at the time of valve replacement (mean LVEF 52 ± 12%), while the population of the latter study mainly had a preserved LVEF (61.5 ± 14.5%). Assignment to a specific hemodynamically defined AS subgroup was not performed and could explain the observed discrepancies. In our population, a significant increase of LVEF could only be observed for the LF/LG group, whereas LVEF in the other subgroups was preserved. This recovery of left-ventricular function was paralleled by a significant increase in longitudinal strain and velocity. Hence, there was a significant impairment of longitudinal strain in HF/HG AS patients despite normal LVEF. TAVR resulted in a significant recovery of longitudinal strain in this group as well, leading to similar values as in the control population with no persisting statistically significant difference. Therefore, subtle changes in left-ventricular function can be detected by CMR strain imaging.

Findings of recent studies suggest that in patients with PLF/LG AS, a more advanced left-ventricular remodeling in comparison to other AS subgroups can be documented. It could be demonstrated that in this specific subset, a more pronounced deposition of fibrotic tissue occurs [35, 36]. This limits the potential for positive remodeling after valve replacement. In concordance with this concept, we did not observe an improvement in strain parameters or NT-proBNP levels for this particular subgroup. This finding is in line with observational data suggesting greater benefit from TAVR for HF/HG patients in comparison to PLF/LG patients [39–41]. Nevertheless, even though benefit is lower for PLF/LG patients in comparison to other AS subgroups, a clinical improvement could also be observed for these particular patients. It could be assumed that—even without improvement of neither longitudinal strain nor LVEF—reduction of left-ventricular afterload leads to a consecutive reduction of left-ventricular end-diastolic pressure and post-capillary pulmonary hypertension. This fact may explain the observation that PLF/LG patients also do better with aortic valve replacement than with conservative treatment [39–41]. The findings of our study, however, provide a rationale for investigating the optimal timing for an aortic valve intervention in case of a PLF/LG situation. Current guidelines recommend aortic valve replacement by the time symptoms occur or if LVEF is decreased below 50% in asymptomatic patients [2, 5]. This concept has been challenged, especially with regard to the PLF/LG situation. The limited clinical benefit in addition to unchanged pathologic strain imaging results supports the idea that this AS subgroup might benefit from an intervention earlier in the course of the disease when the potential for reverse remodeling may be greater [42]. Therefore, it has been suggested to expand the diagnostic work-up of AS patients beyond evaluation of LVEF and symptoms and to perform a more detailed evaluation of left-ventricular function and remodeling. The assessment of cardiac mechanics by CMR strain imaging may facilitate the optimal timing for treatment and the prediction of recovery. Of course, the hypothesis generated from these considerations would have to be proven in prospective randomized trials, in which strain assessment by CMR could be used as beneficial surrogate.

Tissue tracking technologies for CMR that enable strain assessment on standard cine sequences have been emerging over the last years. They already could prove their usefulness in various clinical settings and are thought to provide deeper insights in cardiac (dys-) function [23]. Especially longitudinal strain is an early marker of impaired left-ventricular function. Reason for this finding is the fact that longitudinal function mainly is provided by subendocardial fibers [43]. This is of special interest in the setting of severe AS, since a subendocardial dysfunction is likely to occur in the presence of an increased left-ventricular afterload, even in early stages of the disease. We were able to support this assumption in our study and favor further assessment and evaluation of this parameter in AS patients.

## Limitations

Several limitations need to be addressed. In our study, only longitudinal strain showed a strong correlation to the hemodynamic situation. Our results were in line with other studies proving the relevance of longitudinal strain in AS patients, even though most of the studies forming the evidence base use echocardiographic derived strain [44]. For radial and circumferential strain, values throughout the study population varied to a much greater extend. This finding might be explained by concomitant cardiac diseases, e.g. coronary artery disease that mainly affects regional left-ventricular function. Form and extent of coronary artery disease have a relevant impact on strain measurements [45]. However, it most probably also has an important impact on the way, the left ventricle adapts to AS, as well (e.g. patients with LF/LG mostly exhibit dilated left ventricles and poor ejection fractions due to coronary artery disease). Therefore, we believe that myocardial deformation assessment is a valid tool for the evaluation of left ventricular function in AS patients, irrespective of coronary artery disease. Eventually, critical coronary stenosis (e.g. left-main) usually was revascularized several weeks before AVR.

Though our study cohort is relatively small, we were able to provide strain values for the distinct AS subgroups. These values are modality and software specific and are not simply transferable to other settings. The comparability throughout different vendors, imaging modalities and post-processing software needs to be further evaluated and proven.

## Conclusion

Strain imaging by FT CMR strongly correlates to the hemodynamic situation in patients with severe AS and is able to predict remodeling after TAVR.
